# Autologous peripheral blood-derived stem cells transplantation for treatment of no-option angiitis-induced critical limb ischemia: 10-year management experience

**DOI:** 10.1186/s13287-020-01981-4

**Published:** 2020-10-28

**Authors:** Gang Fang, Xiaolang Jiang, Yuan Fang, Tianyue Pan, Hao Liu, Bichen Ren, Zheng Wei, Shiyang Gu, Bin Chen, Junhao Jiang, Yun Shi, Daqiao Guo, Peng Liu, Weiguo Fu, Zhihui Dong

**Affiliations:** 1 Department of Vascular Surgery, Zhongshan Hospital, Fudan University, Shanghai, China; 2 Department of Hematology, Zhongshan Hospital, Fudan University, Shanghai, China; 3 Department of Vascular Surgery, Institute of Vascular Surgery, Zhongshan Hospital, Fudan University, 180 Fenglin Road, Shanghai, 200032 China

**Keywords:** Stem cells transplantation, Critical limb ischemia, Thromboangiitis obliterans, Periphreal blood mononuclear cells, CD34+ cells, Limb salvage

## Abstract

**Background:**

Previous studies have demonstrated that no-option angiitis-induced critical limb ischemia (NO-AICLI) could be significantly improved by transplantation of peripheral blood-derived stem cells (PBDSCs). Additionally, a randomized controlled trial (RCT) recently conducted by us suggested that peripheral blood-derived purified CD34+ cells (PCCs) were not inferior to non-purified peripheral blood mononuclear cells (PBMNCs) at limb salvage in treatment of NO-AICLI. However, most of these clinical trials whether RCT or single-arm studies were characterized with a small sample size and absence of long-term outcomes.

**Methods:**

To analyze long-term clinical outcomes of PBDSCs transplantation for NO-AICLI, we reviewed clinical data of patients with NO-AICLI receiving PBDSCs transplantation at our center during the past decade. Meanwhile, we first compared the long-term safety and efficacy of intramuscular transplantation of PCCs versus PBMNCs in a sizable number of patients with NO-AICLI.

**Results:**

From May 2009 to December 2019, a total of 160 patients with NO-AICLI patients were treated by PBDSCs transplantation (82 with PCCs, 78 with PBMNCs) at our center. Baseline characteristics between two groups were similar. Up to June 2020, the mean follow-up period was 46.6 ± 35.3 months. No critical adverse events were observed in either group. There was one death during the follow-up period. A total of eight major amputations occurred. The cumulative major amputation-free survival (MAFS) rate at 5 years after PBDSCs transplantation was 94.4%, without difference between two groups (*P* = .855). Wound healing, rest pain, pain-free walking time, ankle-brachial index, transcutaneous oxygen pressure, and quality of life (QoL) also significantly improved after PBDSCs transplantation.

**Conclusions:**

Autologous PBDSCs intramuscular transplantation could significantly decrease the major amputation rates and improve the QoL in patients with NO-AICLI. Long-term observation of a large sample of patients confirmed that the clinical benefits of PBDSCs transplantation were durable, without difference between the PCCs and PBMNCs groups.

## Background

Critical limb ischemia (CLI), due to advanced peripheral artery disease (PAD), is characterized with high mortality and major amputation rates [1, 2]. Surgical and endovascular revascularization are the most common used techniques to improve symptoms in patients at this stage, especially for atherosclerosis (ASO)-induced CLI.

However, up to 20% of CLI patients are not eligible for revascularization (no-option CLI) [3, 4] and reported to have 6-month major amputation rates ranging from 10% to 40% [2, 5]. Among patients with angiitis-induced CLI (AICLI), the proportion of “No-option” ones is much higher considering unfavorable anatomical conditions for traditional revascularization techniques and high re-occlusion rates after interventions due to the chronic autoimmune angiitis. Meanwhile, thromboangiitis obliterans (TAO) was the main cause of no-option AICLI (NO-AICLI). Compared with ASO, patients with TAO-induced CLI are remarkably younger and have much higher life expectations, posing a more urgent need for limb salvage and better quality of life (QoL) [6, 7]. However, despite of the great development on endovascular treatment for limb ischemia in recent years, these innovative techniques and equipment are still based on the conventional theory of revascularization, which brings a fairly limited improvement on the poor prognosis of NO-AICLI. New treatment modalities are urgently needed for NO-AICLI.

Autologous stem cell therapy (SCT) aiming at promoting angiogenesis and neovascularization has recently been reported as a safe and effective way for treatment of no-option CLI (NO-CLI) [8–10]. Bone marrow-derived stem cells (BMDSCs) supposed to contain a higher concentration of endothelial progenitor cells was initially used [11–14]. Afterwards, given that obtainment of peripheral blood-derived stem cells (PBDSCs) is less invasive and more convenient, several clinical trials on PBDSCs transplantation for NO-CLI have been conducted and demonstrated comparable outcomes with BMDSCs [15–18]. Our previous pilot study indicated that granulocyte colony-stimulating factor (G-CSF)-mobilized peripheral blood-derived purified CD34+ cells (PCCs) transplantation could achieve favorable outcomes in NO-AICLI (a five-year major amputation-free survival rate (MAFS) of 88.9%) [19, 20]. To investigate whether the angiogenesis induced by PCCs was impaired due to the cell loss during PCCs isolation, we then conducted a randomized controlled trial (RCT) to compare the non-purified peripheral blood mononuclear cells (PBMNCs) with PCCs in the treatment of NO-AICLI. Outcomes revealed that transplantation of both types of cells could significantly improve MAFS compared with conventional therapies and PCCs was not inferior to PBMNCs in the treatment of AICLI [21]. However, despite this, the sample size of this RCT is small and the follow-up period is too short to get long-term results.

The aim of this study was to analyze the short-term clinical efficacy and long-term clinical outcomes of SCT for NO-AICLI at our center during the past decade. Meanwhile, we compared the safety and efficacy of intramuscular transplantation of autologous peripheral blood-derived PCCs versus PBMNCs in a sizable number of patients with NO-AICLI.

## Methods

### Study population

From May 2009 to December 2019, patients with NO-AICLI who received PBDSCs transplantation at our center were consecutively enrolled into this retrospective study. Inclusion criteria for PBDSCs transplantation comprised (1) patients aged 18 to 80 years; (2) presence of stenotic or occlusive lesions in the limb arteries, as confirmed by computed tomography angiography, magnetic resonance angiography, or digital subtraction angiography; (3) CLI with a Rutherford class of 4–5 that was anatomically unsuitable for open surgery or endovascular therapy; (4) no improvement for at least 3 months after open surgery or endovascular treatment, or no alleviation of rest pain after at least 1 month of conservative treatment including smoking cessation, regular drug therapy, dietary control, and exercise therapy; and (5) if present, an unhealing ulcer after at least 1 month of optimal care by a wound care physician and a nurse. Each enrolled patient should meet all of these criteria. Exclusion criteria included (1) serious health events occurred < 3 months before admission, including but not limited to myocardial infarction, cerebral apoplexy, pulmonary embolism, severe hepatic dysfunction, and renal dysfunction or (2) contraindications for the administration of G-CSF. The study complied with the Declaration of Helsinki guidelines and was approved by the Committee for the protection of Human Subjects at Zhongshan Hospital, Fudan University. All patients participating in the study signed an informed consent document.

### Treatment procedures and follow-up protocol

After admission, G-CSF (Neupogen®; Amgen, Thousand Oaks, CA, USA) was injected subcutaneously to mobilize the bone marrow cells for 5 days. Meanwhile, enoxaparin (4000 IU/day) was administered daily to prevent hypercoagulability, and blood test was performed daily to test for white blood cell (WBC) and CD34+ cell counts. Suspension of PBMNCs was collected via leukapheresis (COM.TEC; Fresenius Hemocare GmbH, Bad Homburg, Germany) when the WBC count was elevated > 60 × 10^9^/L or on the fifth day. And for patients in the PCCs group, the suspension was purified by a magnetic cell storing system (MiltenyiBiotec GmbH, BergischGladbach, Germany) before transplantation. Leukocyte counting and flow cytometry was used to test the final cell product. The cell transplantation was performed by intramuscular injection under general anesthesia for the upper extremities or spinal anesthesia for the lower extremities. A total of 80 and 160 sites were each injected with 0.5 mL for patients in the PCCs and PBMNCs groups, respectively. And the injections were distributed in the calf and foot or the forearm and hand of the ischemic limbs evenly. And the final transplanted CD34+ cells were controlled ranging from 10^5^ to 10^6^ per kilogram body weight. All patients were followed once a month for the first 3 months, every 3 months for the rest of the year, and once a year afterwards. Aspirin (100 mg/d), cilostazol (400 mg/d), and anplag (300 mg/d) were administrated for at least 1 year. Major amputations (above the ankle) during the follow-up period and any adverse events related to the treatment procedure were recorded. Ulcer healing, confirmed by a clinician who observed and photographed the patients’ limbs, and Rutherford classification were recorded at the baseline and each follow-up point. Wong-Baker faces pain rating scale (WBFPS), in which a score of 0 represents no pain and a score of 10 represents greatest pain, was used to evaluate the patients’ rest pain in the absence of analgesic agents at the baseline and each follow-up point. The pain-free walking time (PFWT) at 2.5 km/h and at a 10% incline on a treadmill was evaluated 3 and 6 months after transplantation and yearly afterwards. For patients who could not perform the test, the PFWP was recorded as 0. Ankle-brachial index (ABI) and transcutaneous pressure of oxygen (TcPO_2_) of the dorsum were also detected at each follow-up point, and the QoL was evaluated by 36-item Short Form Health Survey (SFHS-36) at the baseline and once a year after cell transplantation. Ophthalmoscopy was used to assess pathological angiogenesis in the retina at each follow-up point.

### Data collection

Prospectively collected clinical data was retrospectively analyzed in the current study. The data included the demographic information, the etiology of the limb ischemia, the risk factors for peripheral artery and cardiovascular diseases, and previous surgical therapy. Treatment information including cell type, characteristics of cell products, and adverse events were also reviewed. Furthermore, the baseline and follow-up features of the treated limb were reviewed, including the Rutherford class, wound healing, ABI, and TcPO_2_ of the dorsum. The WBFPS, PFWT, and QoL of the enrolled patients were also analyzed in this study.

The primary endpoint was the rate of freedom from major amputations of the treated limb. The secondary endpoints included all-cause death, the ulcer healing rate, WBFPS, and PFWT.

### Statistical analysis

Continuous variables were presented as mean ± standard deviation, and categorical variables were presented as frequencies and percentages. The efficacy parameters were analyzed by using a generalized estimating equation model with longitudinal analysis of data changing from the baseline to months 1, 3, 6, 12, 36, and 60. The MAFS was estimated with the Kaplan-Meier method, and differences in survival among the groups were analyzed with the log-rank test. The Wilcoxon signed-rank test was used to compare the QoL values at baseline and each follow-up examination. A *P* value < .05 was considered to indicate a statistically significant difference. The statistical analyses were performed using SAS software, version 9.3 (SAS Institute, Cary, NY).

## Results

### Study population

From May 2009 to December 2019, a total of 172 patients with CLI received SCT at our center. Among them, 12 patients with ASO as their etiology were excluded from this study. Finally, 160 patients with NO-AICLI were enrolled in this study. According to the type of PBDSCs, these patients could be divided into two groups. Eighty-two patients received intramuscular transplantation of PCCs, while 78 patients were treated with PBMNCs.

Baseline patient characteristics are presented in Table 1. TAO was the most common (155/160, 96.9%) cause of limb ischemia in enrolled patients. Patients with TAO were all male and most of them had high frequencies of smoking history. All of the patients presented NO-CLI in one limb, except for 4 patients who had more than 2 ischemic limbs, the most ischemic of which, those with ABI < 0.4, were included in the current study. Rutherford 5 patients accounted for 89.4% at enrollment. Most of patients underwent regular medical therapy and/or at least one type of revascularization techniques before PBDSCs transplantation. The baseline characteristics were similar between two cell groups.

**Table 1:** Baseline characteristics of enrolled patients

Characteristics	Total	PCCs (82)	PBMNCs (78)	*P*
Age, years	41.2 (11.2)	40.6 (10.5)	41.8 (11.8)	.610
Male, *n* (%)	157 (98.1)	80 (97.6)	77 (96.3)	.990
BMI, kg/m^2^	23.2 (3.7)	23.5 (3.5)	22.9 (3.8)	.820
Etiology				
TAO, *n* (%)	155 (96.9)	79 (96.3)	76 (97.4)	1.000
Eosinophilia, *n* (%)	1 (0.63)	0 (0)	1 (1.30)	.487
SLE, *n* (%)	3 (1.88)	2 (2.4)	1 (1.3)	1.000
Erythema nodosum, *n* (%)	1 (0.63)	1 (1.2)	0 (0)	1.000
Comorbidities				
Smoking, *n* (%)	145 (90.6)	72 (87.8)	73 (93.6)	.210
Hypertension, *n* (%)	8 (5.0)	5 (6.1)	3 (3.8)	.720
Type II diabetes mellitus, *n* (%)	8 (5.0)	4 (4.9)	4 (5.1)	.990
Hyperlipidemia, *n* (%)	11 (6.88)	7 (8.5)	4 (5.1)	.394
Coronary artery disease, *n* (%)	3 (1.88)	2 (2.4)	1 (1.3)	.990
Treated limbs				
Upper/lower extremity, *n*	5/155	3/79	2/76	.990
Right/left, *n*	86/74	44/38	42/36	.989
Ulcer only, *n* (%)	75 (46.9)	34 (41.5)	41 (52.6)	.160
Gangrene, *n* (%)	70 (43.8)	37 (4.1)	33 (42.3)	.720
Rutherford class				
4, *n* (%)	15 (18.3)	11 (13.4)	4 (5.1)	.720
5, *n* (%)	145 (90.6)	71 (86.6)	74 (94.9)	.720
Medication history				
Aspirin, *n* (%)	134 (83.8)	66 (80.5)	68 (87.2)	.251
Clopidogrel, *n* (%)	18 (11.3)	10 (12.2)	8 (11.2)	.698
Cilostazol, *n* (%)	61 (38.1)	32 (39.0)	29 (37.2)	.810
Prostaglandins, *n* (%)	124 (77.5)	61 (74.4)	63 (80.8)	.334
Warfarin, *n* (%)	6 (3.8)	4 (4.9)	2 (2.6)	.682
Rivaroxaban, *n* (%)	5 (3.1)	2 (2.4)	3 (3.8)	.676
Surgical history				
Bypass, *n* (%)	18 (11.3)	11 (13.4)	7 (9.0)	.374
Endarterectomy, *n* (%)	5 (3.1)	3 (3.7)	2 (2.6)	1.000
PTA, *n* (%)	42 (26.3)	20 (24.2)	22 (28.2)	.584
Stenting, *n* (%)	16 (10.0)	8 (9.8)	8 (10.3)	.916
Thrombolysis, *n* (%)	52 (32.5)	28 (34.1)	24 (30.8)	.648
Sympathectomy, *n* (%)	7 (4.4)	2 (2.4)	5 (6.4)	.268
Surgical thrombectomy, *n* (%)	22 (13.8)	12 (14.6)	10 (12.8)	.739
Excimer laser thrombectomy, *n* (%)	7 (4.4)	2 (2.4)	5 (6.4)	.268

*PCCs* purified CD34+ cells, *PBMNCs* peripheral blood mononuclear cells, *BMI* body mass index, *TAO* thromboangiitis obliterans, *SLE* systemic lupus erythematosus, *PTA* percutaneous transluminal angioplasty

**Fig. 1:**
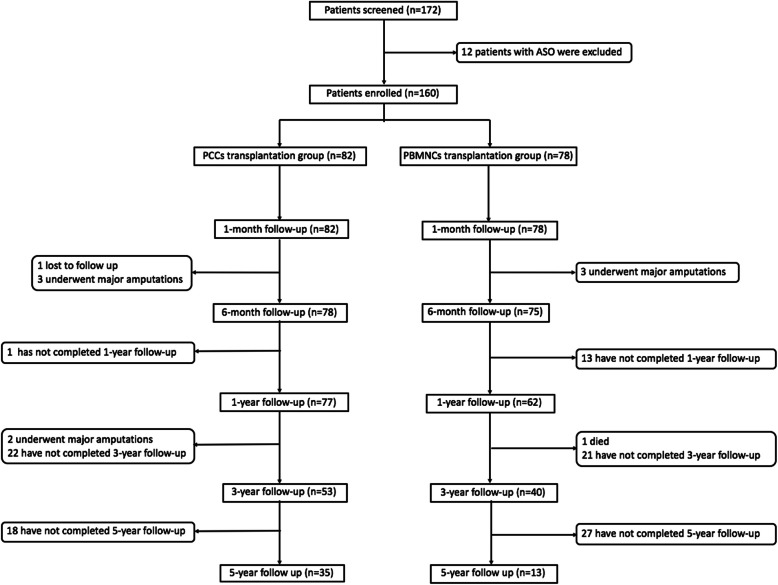
Trial profile. ASO, atherosclerosis, PCCs, purified CD34+ cells; PBMNCs, peripheral blood mononuclear cells

Up to June 2020, the mean follow-up period was 46.6 ± 35.3 months (range 6–132 months). There were eight major amputations during follow-up. One patient in the PCCs group was lost to follow-up at 2 months and one patient in the PBMNCs group died at 18 months. A total of 48 patients have completed the 5-year follow-up evaluation. The specific follow-up information is shown in Fig. 1.

### Quality of the cell products

The mean final transplant volumes were 39.2 mL in the PCCs group and 85.4 mL in the PBMNCs group (*P* < .001). The total WBC counts and concentrations were significantly higher in the PBMNCs group than in the PCCs group (*P* < .001). There was no significant difference in CD34+ cell concentrations between two groups (*P* = .843, Table 2).

**Table 2:** Characteristics of the cell product

Characteristics	PCCs(82)	PBMNCs(78)	*P*
Final volume, ml	39.2 (1.3)	85.4 (22.7)	< .001
Total WBC count, ×10^6^	53.1 (25.6)	21,400 (11300)	< .001
WBC concentration, ×10^9^/L	1.53 (0.72)	249 (121)	< .001
CD34+ cell concentration, × 10^8^/L	8.15 (4.23)	8.24 (4.11)	.843
CD34+ cells/WBCs, %	65 (23)	0.33 (0.15)	< .001

*PCCs* purified CD34+ cells, *PBMNCs* peripheral blood mononuclear cells, *WBCs* white blood cells

### Safety evaluation

There were no critical adverse events during the treatment and follow-up period. The counts of WBC decreased to normal level within 1 week in all patients after transplantation. Fifteen patients suffered adverse events during the mobilization period, including 1 patient with slight fever, 3 with back pain, 2 with transient headache, and 2 with pruritus in the PBMNCs group and 3 patients with slight fever, 2 with back pain, and 2 with pruritus in the PCCs group. All of them completely recovered within 2 weeks after transplantation. Pathological retinal angiogenesis was not observed during the follow-up.

### All-cause death and major amputations

**Fig. 2:**
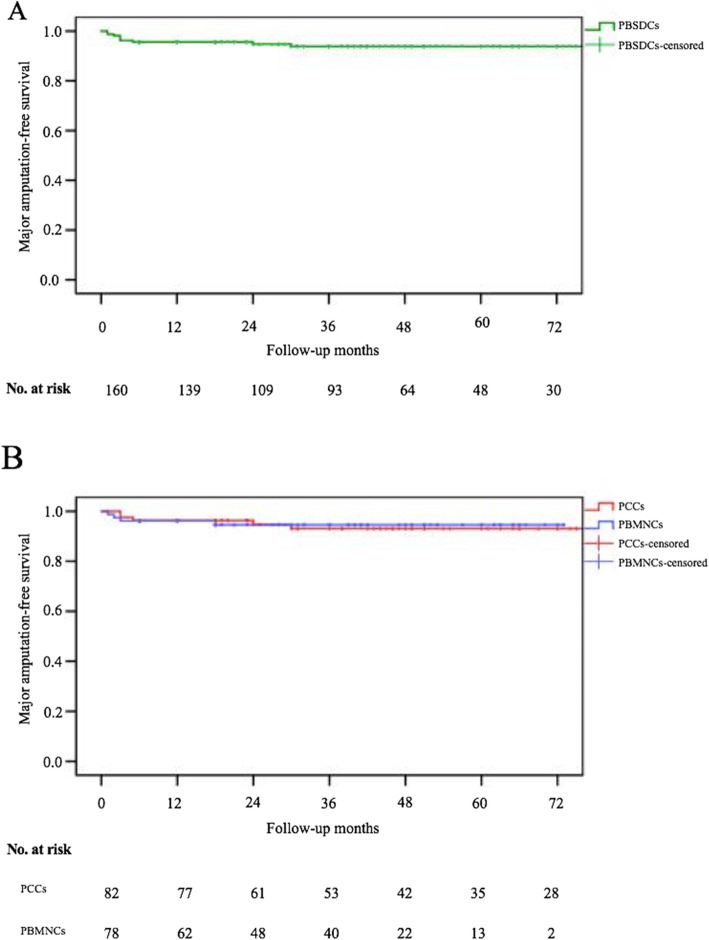
Kaplan-Meier analysis of major amputation-free survival rates after PBDSCs transplantation (**a**). Kaplan-Meier analysis of major amputation-free survival rates for the PCCs versus PBMNCs groups (**b**). PBDSCs, peripheral blood-derived stem cells; PCCs, purified CD34+ cells; PBMNCs, peripheral blood mononuclear cells

There was one death during the follow-up period. One patient in the PBMNCs group died at 18 months after SCT. The death was identified as not related to the study interventions. A total of eight major amputations occurred within the mean follow-up period of 46.6 ± 35.3 months. There were five major amputations in the PCCs group: two occurred at 3 months, the remaining three amputations at 5, 24, and 30 months, respectively. Three major amputations occurred in the PBMNCs group at 1, 2, and 3 months, respectively. The cumulative MAFS rate at 5 years after PBDSCs transplantation was 94.4%. The 5-year MAFS rates were 93.9% in the PCCs group and 94.9% in the PBMNCs group (*P* = .855, Fig. 2). When evaluating the major amputation rate by Rutherford classification in all patients, all major amputations occurred in Rutherford 5 patients, no enrolled Rutherford 4 patients underwent any major amputations. However, the difference was not statistically significant (5.5% vs 0, *P* = 1.0).

### Efficacy evaluation

**Fig. 3:**
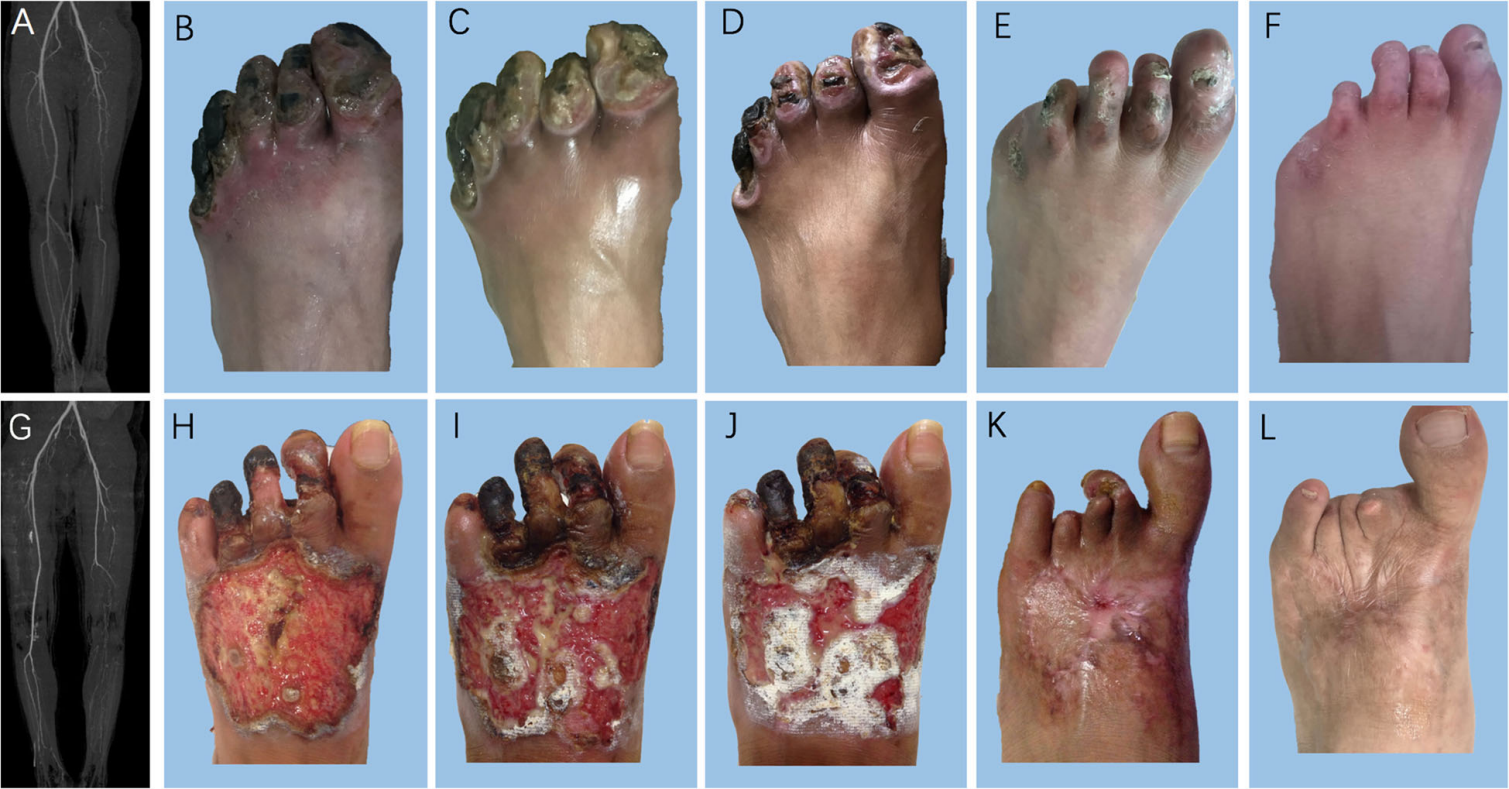
Wound healing in the PCCs and PBMNCs groups. In a 37-year-old male with TAO (**a**–**f**), preoperative CTA showed artery occlusion on the left lower extremity with the occlusion level at the orifice of the superficial femoral artery (**a**). The patient had gangrene and ulcers on his left toes before PCCs transplantation (**b**). Wound was gradually healing at 1 (**c**), 3 (**d**), and 6 (**e**) months after PCCs transplantation and complete wound healing was observed at 12 months (**f**). In a 23-year-old male with TAO (**g**–**l**), preoperative CTA showed artery occlusion on the left lower extremity with the occlusion level at the distal superficial femoral artery (**a**). The patient had gangrene on his left toes and a large ulcer on the dorsum of his left foot before PBMNCs transplantation (**b**). Wound was gradually healing at 1 (**c**), 3 (**d**), and 6 (**e**) months after PBMNCs transplantation and complete wound healing was observed at 12 months (**f**). PCCs, purified CD34+ cells; PBMNCs, peripheral blood mononuclear cells, TAO, thromboangiitis obliterans

There were 71 (86.6%) and 74 (93.7%) patients with tissue loss in the PCCs and PBMNCs group, respectively. A total of 140 patients with tissue loss completed a 6-month follow-up (Fig. 3). Complete wound healing at 6 months was observed in over 60% of patients. In patients completing the 5-year follow-up, complete wound healing occurred in all 47 patients with tissue loss at enrollment, without difference between the two groups. Rutherford stage also gradually improved in both groups after SCT. All enrolled patients were defined as Rutherford 4 or 5 class of limb ischemia. After a 6-month follow-up, approximately 58% of patients had an obvious improvement on Rutherford stage to non-CLI. Notably, the proportion of non-CLI in patients completing 3-year follow-up reached over 93% and persisted till the 5-year follow-up, without difference between the two groups (Table 3).

**Table 3:** Wound healing, and Rutherford class 0–3 at 1, 6, 12, 36, and 60 months

Outcome	Total	PCCs	PBMNCs	*P*
Complete wound healing, *n* (%)				
At enrollment	0/145	0/71	0/74	1.000
At 1 months	13/145	6/71	7/74	.846
At 6 months	85/140	40/69	45/71	.512
At 12 months	111/128	58/68	53/60	.613
At 36 months	86/90	50/51	36/39	.880
At 60 months	47/47	34/34	13/13	.292
Rutherford class 0–3, *n* (%)				
At enrollment	0/160	0/82	0/78	1.000
At 1 month	19/160	10/82	9/78	.898
At 6 months	89/153	43/78	46/75	.437
At 12 months	120/139	64/77	56/62	.219
At 36 months	87/93	50/53	37/40	1.000
At 60 months	47/48	34/35	13/13	1.000

*PCCs* purified CD34+ cells, *PBMNCs* peripheral blood mononuclear cells

**Fig. 4:**
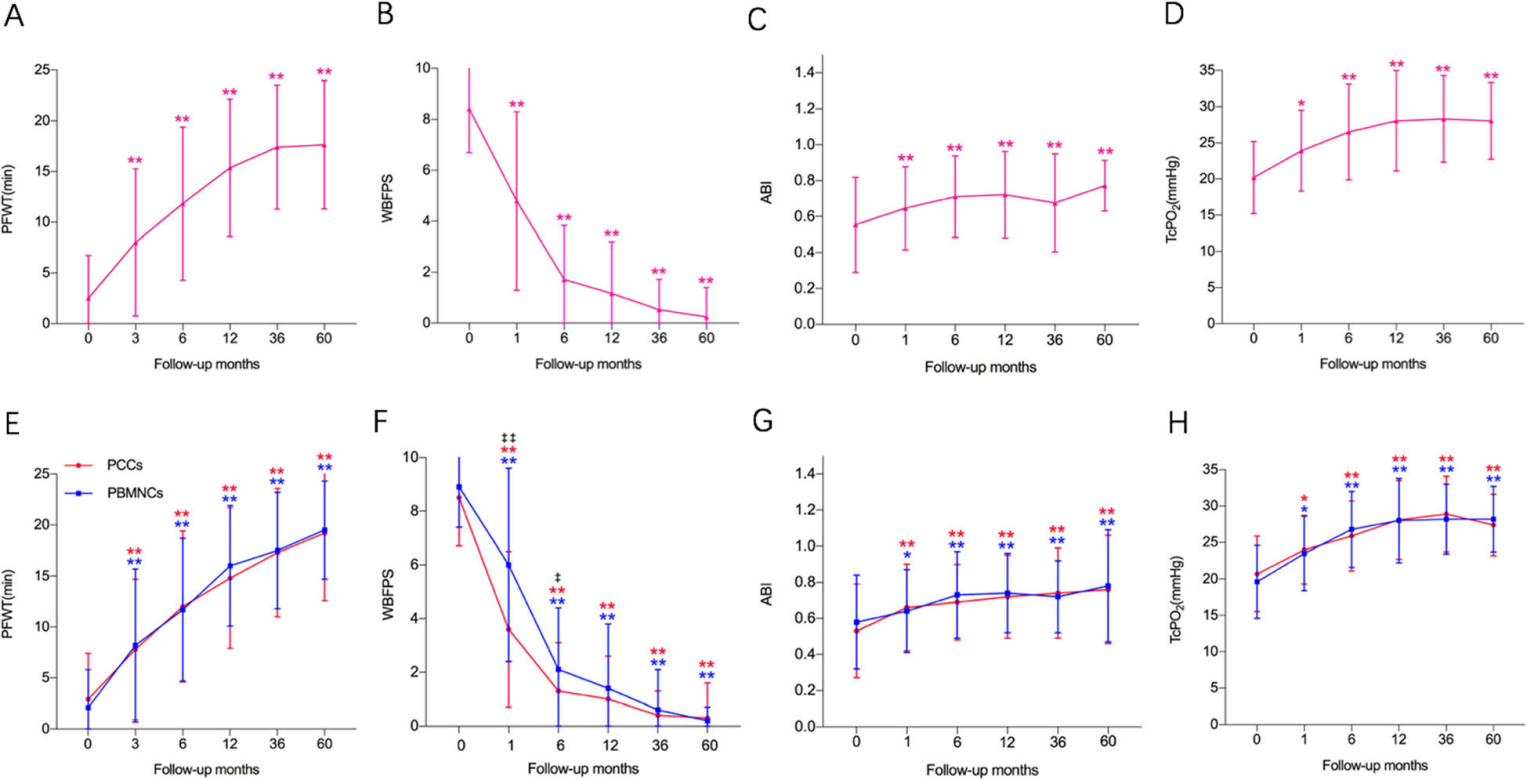
Serial changes in PFWT (**a**), WBFPS (**b**), ABI (**c**), and TcPO_2_ (**d**) after PBDSCs transplantation. Comparison of the serial changes in PFWT (**e**), WBFPS (**f**), ABI (**g**), and TcPO_2_ (**h**) between the PCCs and PBMNCs groups. PBDSCs, peripheral blood-derived stem cells. PCCs, purified CD34+ cells; PBMNCs, peripheral blood mononuclear cells, PFWT, pain-free walking time; WBFPS, Wong-Baker faces pain rating scale; ABI, ankle-brachial index; TcPO_2_, transcutaneous pressure of oxygen. *P < .05 compared with the baseline; **P < .01 compared with the baseline; ^‡^*P* < .05 compared between two groups; ^‡‡^*P* < .01 compared between two groups

The WBFPS and PFWT values after SCT gradually improved and this improvement persisted till 5 years. The mean values of WBFPS and PFWT were significantly better at each follow-up visit compared with baseline measurements. No difference was observed in both two parameters at each follow-up examination between the PCCs and PBMNCs groups except that the WBFPS values at 1 and 6 months were significantly lower in the PCCs group (Fig. 4).

Regarding to perfusion evaluating parameters, mean ABI and TcPO_2_ values were comparable between the two groups before treatment, and they improved significantly at each follow-up visit compared with the baseline. In addition, no difference in ABI and TcPO_2_ was observed between the PCCs and PBMNCs groups before and after SCT (Fig. 4).

**Fig. 5:**
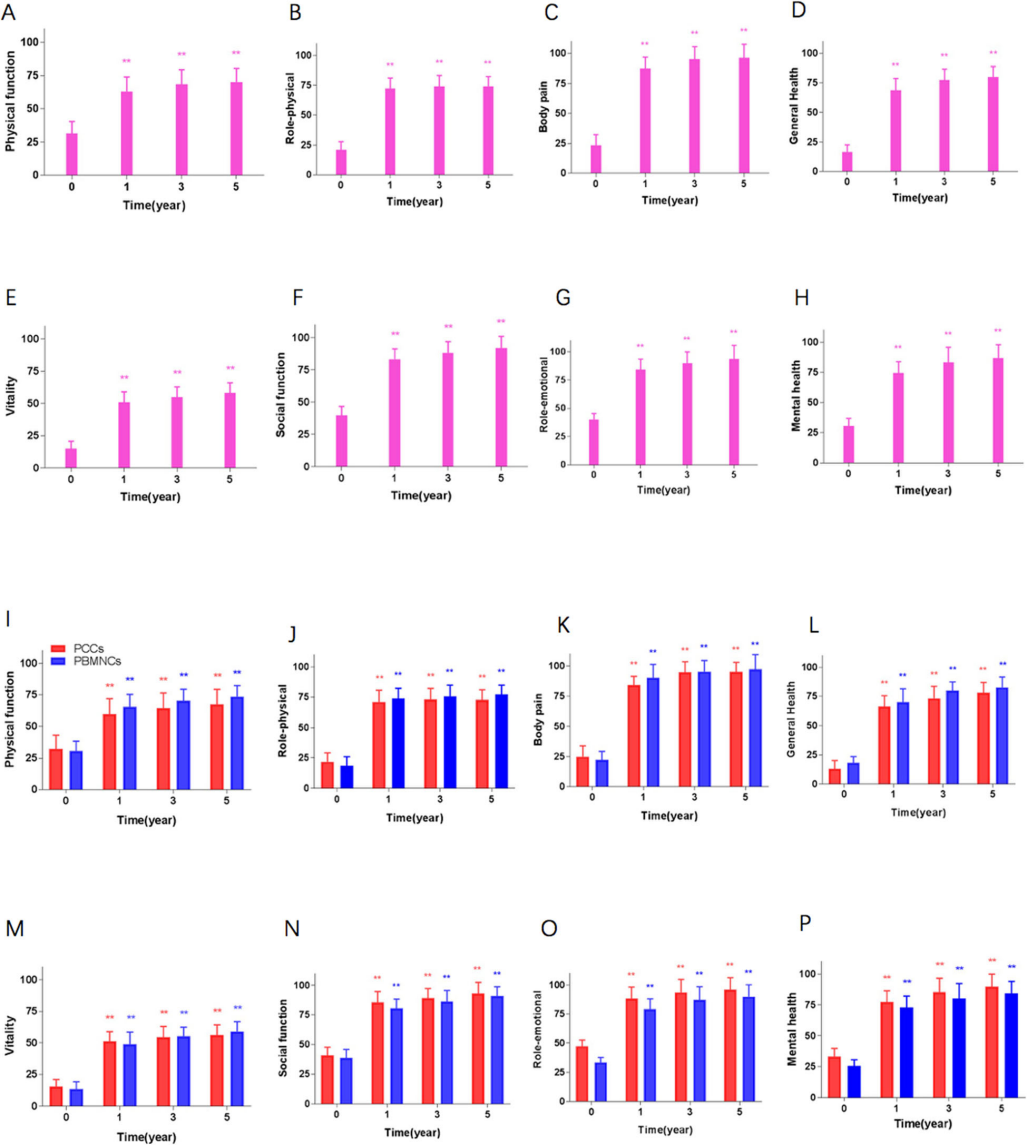
Assessment of the quality of life using the SFHS-36 after PBDSCs transplantation (**a**–**h**). Comparison of the quality of life using the SFHS-36 between the PCCs and PBMNCs groups (**i**–**p**). There are 8 dimensions in the SFHS-36: physical function, role-physical, body pain, general health, vitality, social function, role-emotional, and mental health. PCCs, purified CD34+ cells; PBMNCs, peripheral blood mononuclear cells; SFHS-36, 36-item Short Form Health Survey. **P < .01 compared with the baseline

QoL scores gradually increased after SCT and persisted till 5 years. There was a significant improvement in all eight sections of SFHS-36 at 1, 3, and 5 years compared with the baseline, without difference between the PCCs and PBMNCs groups (Fig. 5).

## Discussion

Although not as common as ASO-induced CLI, AICLI still represents an important subgroup of advanced PAD. Meanwhile, AICLI is characterized with higher incidence in East Asians, much lower onset age, and a higher proportion of no-option ones, which causes a tricky dilemma that conventional revascularization modalities hardly work in these patients with higher levels of expectation of limb salvage, especially in China where there is a large population of patients with AICLI [22, 23]. This is one of the reasons why we selected patients with NO-AICLI as our treatment subjects by SCT. Several previous studies have demonstrated PBDSCs transplantation presented similar outcomes with BMDSCs on limb salvage [18, 24, 25]. However, the sample size was relatively small whether in RCT or single-arm studies [26–29]. To our knowledge, the current series represents the largest number of patients with AICLI treated by PBDSCs transplantation in a single center. Meanwhile, though not designed as an RCT, the baseline characteristics of patients in PCCs and PBMNCs groups are similar in the current retrospective study, which therefore ensures the comparability of outcomes after transplantation of two types of PBDSCs. This study shows that autologous PBDSCs intramuscular transplantation was effective in improving symptoms at an early stage and the clinical efficacy persisted over the long term, without any serious adverse events. A significant improvement on clinical and hemodynamic outcomes was observed in both treatment groups without any significant difference between the groups, validating that the clinical efficacy of PCCs was not impaired due to the cell loss during PCCs isolation. Based on the outcomes of the current study and the fact that CD34+ cells are the key component of endothelial progenitor cells-enriched fraction of PBMNCs, we believe that CD34+ cells play a predominant role in PBMNCs transplantation for NO-AICLI. Despite this, the PCCs group with obviously higher purity of CD34+ cells did not present better clinical efficacy compared with the PBMNCs group. Hence, it remains to be investigated whether the therapeutic effects of PBDSCs transplantation could be further improved.

Several previous clinical studies have demonstrated the beneficial effect of SCT on limb salvage and ulcer healing in NO-AICLI compared with placebo intervention [20, 21, 30–32]. More recent data reported by Gu et al. suggested that the 10-year amputation-free survival was 85.3% in patients with TAO treated by BMMNCs, similar with the long-term MAFS in this study [33]. However, such favorable limb salvage rates were not observed in ASO-induced NO-CLI by SCT. More recent studies observed that patients with NO-AICLI benefit much more from SCT than those with ASO-induced NO-CLI [19, 34]. The JUVENTAS trial, which was deemed as a well-designed double-blinded, placebo-controlled clinical trial with a relatively larger sample size, enrolled patients with ASO-induced NO-ACL as the majority of the subjects [35]. Outcomes of this study indicated autologous BMDSCs repetitive intra-vascular transplantation had no effect on the primary outcome of major amputation rates compared with the placebo group (19% vs 13%) [36]. The failure in validating the beneficial effects of BMDSCs administration in the JUVENTAS trial was partly attributed to the high proportion of enrolled aged patients with a high systemic atherosclerotic burden and prevalence of cardiovascular disease. Moreover, our recent published study has reported that age ≥ 50 years and arterial occlusion above the knee/elbow combined with blood fibrinogen, TcPO_2_, and the total transplanted CD34+ cell count were independent prognostic factors of the responders to PBDSCs -based therapeutic angiogenesis for NO-CLI, which suggested that ASO-induced NO-CLI characterized with an advanced age and a higher arterial occlusion plane was less likely to benefit from SCT [37]. Therefore, despite that the first published clinical trial on SCT for NO-CLI conducted by Tateishi-Yuyama et al. enrolled patients with ASO as the major group of subjects, NO-AICLI seemed to be the better candidate in advanced PAD for SCT according to published data.

An interesting observation in the current study was that all major amputations occurred in Rutherford 5 patients. Despite equally classified into CLI and enrolled as an important subgroup in the present study, Rutherford 4 patients without tissue loss were at a much lower risk of major amputations after SCT than Rutherford 5 patients. Similar observations have been reported in recent years. Benoit et al. conducted a prospective double-blinded RCT (2:1 therapy to control) of 48 patients with NO-CLI treated with BMDSCs and found that amputation rates in patients with tissue loss were significantly higher than patients with rest pain only (46.7% vs 5.6%, *P* = .0029) [38]. A further meta-analysis of the literature was performed and confirmed a difference in amputation rates between patients with tissue loss and rest pain [38]. These outcomes suggested that the number of enrolled Rutherford 4 patients should be limited in a clinical trial regarding SCT for NO-CLI if the primary endpoint of the trial is major amputation rate or MAFS, considering that the much lower major amputation rates in Rutherford 4 patients might dilute the event rate in the overall enrolled population making it difficult to assess the clinical efficacy. In addition, given that the much more favorable outcomes of SCT for AICLI compared with traditional modalities and the significant difference in limb salvage after SCT between patients with rest pain and tissue loss, we highly recommend to use PBDSCs transplantation as the first-choice therapy in patients with angiitis-induced Rutherford 4 class limb ischemia, avoiding that the possibility of progressing into tissue loss during the phase of traditional revascularization treatment which seldom works in AICLI.

As described above, patients with tissue loss correlates a relatively poorer prognosis than patients with rest pain after SCT. Despite this, in the current study, the major amputation rates of 5.5% in patients with tissue loss were still rather encouraging given that these patients were at a huge risk of major amputations with traditional therapies. SCT is characterized with a lower and slow-acting improvement of limb perfusion. Therefore, cell therapy was considered challenging to reverse more acute, diffuse, and critical ischemia. For this reason, Rutherford 6 patients with major tissue loss were excluded from the current study. Almost all clinical trials on SCT for CLI categorized the extent of tissue loss by the Rutherford classification. In studies containing Rutherford 6 patients, Madaric et al. reported an association between Rutherford 6 limb ischemia and a negative therapeutic outcome of SCT, which was in consistent with the results of the PROVASA trial where Rutherford 6 patients at baseline did not benefit from SCT [39, 40]. However, as reported by Mills et al., the Rutherford classification of lower extremity ischemia lacks sufficient detail such like depth of the wound and presence and severity of infection with respect to wound categorization, failing to achieve more precise stratification among patients with tissue loss to aid in selection of the best therapy [41]. The WIFI classification has been regarded as a more objective and accurate classification of the ischemia-induced lower extremity wound based on the degree of ischemia, wound extent, gangrene, and infection, creating a more reasonable gradient of limb perfusion required for wound healing [41]. There is a great potential for application of the WIFI classification in future clinical trials on SCT, as more accurate and detailed stratification of patients with tissue loss would yield a better platform for performing more meaningful comparisons, thereby determining the optimal target population that could benefit from SCT among patients with tissue loss.

The mechanism of improvement of limb ischemia after SCT remains to be investigated. Unlike with mechanical revascularization of relatively larger arteries in conventional open and endovascular interventions, the mechanism of SCT is more complicated and mainly based on angiogenesis by differentiation of transplanted stem cells into endothelial lineage cells composing the structure of micro-circulation [3, 42–44]. Paracrine action and anti-inflammation effects have also recently been demonstrated as important roles in SCT [45–47]. Hemodynamic parameters like ABI and TcPO_2_ were commonly used in evaluating lower limb perfusion and regarded as effective noninvasive modalities correlating well with clinical severity parameters after revascularization interventions [48]. Considering that the functional mechanism of cell therapy is totally different from revascularization modalities, application of conventional hemodynamic parameters, especially ABI which reflects the blood flow in relatively large size of artery, in predicting the clinical trend after SCT remains to be studied [26, 37, 38]. Fujita et al. observed that the time course of the improvement on clinical parameters were not parallel with functional parameters after SCT. It was also reported that the change of ABI after SCT was rather limited despite the great improvement of the clinical status [49]. Similarly, though the mean ABI and TcPO_2_ values in the current study significantly increased compared with the baseline, whereas the degree of change is still modest compared with the dramatic improvement of ischemic symptoms. These results suggested that clinical severity parameters including ulcer healing and Rutherford class might be more valid endpoints for assessing the treatment efficacy than hemodynamic parameters in trials investigating SCT for CLI at least for now. Hence, with SCT more commonly used in limb ischemia, apart from continuing exploring the mechanism of perfusion restoration after SCT, it is also of great significance to find a safe, fast, and easily repeatable hemodynamic testing modality more appropriate for cell therapy to accurately monitor the change of lower extremity perfusion, thereby allowing vascular specialists to refine our current understanding of the disease process while assessing wound healing potential, optimizing the clinical decision making, and improving outcomes after SCT.

The advantages of the present study include its well-designed inclusion criteria for SCT, a larger sample size, comparability of both groups of patients in terms of their baseline characteristics, prospective follow-up, and comprehensive assessments. The main limitation of this study is that the analysis was retrospectively in nature although the data were collected prospectively. In addition, while the mean follow-up period of enrolled patients was relatively longer, the proportion of patients completing a 5-year follow-up was small in the PBMNCs group.

## Conclusions

Autologous PBDSCs intramuscular transplantation could significantly decrease the major amputation rates and improve the QoL in patients with NO-AICLI. Long-term observation of a large sample of patients confirmed that the clinical benefits of PBDSCs transplantation were durable, without difference between the PCCs and PBMNCs groups.

## Data Availability

The datasets used and analyzed during the current study are available from the corresponding author on reasonable request.

## Abbreviations

SCT: Stem cell therapy
PBDSCs: Peripheral blood-derived stem cells
PBMNCs: Peripheral blood mononuclear cells
PCCs: Purified CD34+ cells
NO-AICLI: No-option angiitis-induced critical limb ischemia
AICLI: Angiitis-induced critical limb ischemia
